# CRISPR/Cas9-mediated targeted editing of *GmSH2* enhances sugar accumulation in vegetable soybean

**DOI:** 10.1093/plphys/kiag181

**Published:** 2026-05-18

**Authors:** Cong Li, Baihong Zhang, Jinbao Gu, Yan Lin, Yuhang Zhang, Mingfu Wen, Xiaoyan Liang, Qiangwei Rao, Jin He, Sanwei Yang, Zhen-Yu Wang

**Affiliations:** Institute of Nanfan & Seed Industry, Guangdong Academy of Sciences, Guangdong 510316, China; Zhanjiang Research Center, Institute of Nanfan Seed Industry, Guangdong Academy of Sciences, Guangdong 524300, China; Institute of Nanfan & Seed Industry, Guangdong Academy of Sciences, Guangdong 510316, China; Zhanjiang Research Center, Institute of Nanfan Seed Industry, Guangdong Academy of Sciences, Guangdong 524300, China; Institute of Nanfan & Seed Industry, Guangdong Academy of Sciences, Guangdong 510316, China; Institute of Nanfan & Seed Industry, Guangdong Academy of Sciences, Guangdong 510316, China; Institute of Nanfan & Seed Industry, Guangdong Academy of Sciences, Guangdong 510316, China; Zhanjiang Research Center, Institute of Nanfan Seed Industry, Guangdong Academy of Sciences, Guangdong 524300, China; Institute of Nanfan & Seed Industry, Guangdong Academy of Sciences, Guangdong 510316, China; Institute of Medicinal and Oil Crops, Anshun Academy of Agricultural Sciences, Anshun 561000, China; College of Agriculture, Guizhou University, Guizhou 550025, China; College of Agriculture, Guizhou University, Guizhou 550025, China; Institute of Nanfan & Seed Industry, Guangdong Academy of Sciences, Guangdong 510316, China

## Abstract

Disrupting starch biosynthesis in soybeans redirects seed carbon partitioning toward soluble sugars, offering a genetic strategy to breed sweeter crops.

Dear Editor,

Soybean (*Glycine max*) is a globally important crop cultivated for edible oil, protein, and biodiesel. Additionally, its seeds serve as raw materials for tofu, soy milk, and fermented products (Manavalan et al. 2009). In East Asia and, more recently, worldwide, a distinct type of soybean known as vegetable soybean, marketed as “Mao dou” in China and “edamame” in Japan, has emerged as a premium vegetable crop. These soybeans are harvested at the R6–R7 stage (full seed to beginning maturity) and marketed either as fresh or frozen pods. Their appeal lies in their sweet flavor, plump seeds, bright green pods, and rich nutritional profile, which includes abundant protein, soluble sugars, vitamins, minerals, fiber, and bioactive isoflavonoids (Hu and Lin 2018; Liu et al. 2022). Among these traits, taste quality is of paramount importance, with sweetness being the most critical factor influencing consumer preference. Sweetness is determined primarily by soluble sugar content—over 80% of which is sucrose, followed by fructose and glucose (Zhang et al. 2017; Agyenim-Boateng et al. 2023). Understanding the genetic regulation of sugar metabolism is thus essential for breeding improved vegetable soybean cultivars with superior flavor profiles.

Recent studies have identified several loci associated with sugar content in soybean through genome-wide association study and QTL mapping (Wang et al. 2019; Xu et al. 2022; Hu et al. 2023). For example, a 200-kb interval on chromosome 8 (19,496,314 to 19,698,413 bp) has been linked to seed sucrose content (Xu et al. 2022). However, functional validation of these candidate genes remains limited. *GmSWEET15* knockout causes severe seed abortion and reduces embryo sugar levels (Wang et al. 2019), while the DT1 transcription factor suppresses sucrose transport by interacting with GmSWEET10a (Li et al. 2024). These findings highlight the need for a comprehensive functional dissection of the genes involved in sugar accumulation.

In cereal crops, ADP-glucose pyrophosphorylase (AGPase), a key enzyme in starch biosynthesis, has been extensively studied. In rice, mutation of *OsAGPL2*, which encodes a cytosolic AGPase large subunit, disrupts starch synthesis and leads to sugar accumulation (Tang et al. 2016). In maize, Shrunken2 (Sh2) encodes the AGPase large subunit in endosperm. *sh2* mutants exhibit nearly abolished starch synthesis and accumulate up to 2.5-fold more soluble sugars, forming the genetic basis of supersweet corn (Greene and Hannah 1998; Dong et al. 2019). Supersweet corn now dominates over 70% of the global sweet corn market. However, the role of SH2 homologs in legumes such as soybean remains largely unexplored.

To elucidate the biological function of SH2 homologs in soybean, we characterized GmSH2a and GmSH2b, identified as orthologs of maize *Sh2* containing the conserved NTP-transferase domain essential for ADP-glucose synthesis (Fig. 1a, b). While *GmSH2a* is expressed in flowers, buds, and seeds, *GmSH2b* transcripts are largely confined to the seed (Figure S1). To assess their function without inducing the lethality often associated with complete starch loss, we targeted exon 12 using CRISPR/Cas9 to generate single and double mutants (Fig. 1c; S2a). Consistent with their tissue-specific expression, the *gmsh2a* mutant accumulated excess sugar in leaves, whereas the *gmsh2b* mutant showed higher sugar levels in seeds (Figure S2b, c). Notably, the double mutant exhibited significantly higher sugar accumulation than either single mutant (Figure S2b, c), indicating functional redundancy between *GmSH2a* and *GmSH2b*. Thus, we focused subsequent analyses on the double mutant. Transcriptional analysis confirmed a substantial reduction in *GmSH2a* and *GmSH2b* transcript levels in the double mutant relative to HC6 (Fig. 1d). AGPase activity was also markedly lower in the mutants than that in HC6 (Fig. 1e). Field evaluations confirmed that the mutants maintained wild-type agronomic performance, with no significant penalties in plant height, leaf morphology, seed size, grain plumpness, or yield (Fig. 1f; S3, S4). At the R6–R7 stages, total sugar and sucrose levels were elevated in both leaves and seeds of the mutants, whereas starch content was diminished by approximately 40% relative to HC6 (Fig. 1g, S2). This high-sugar phenotype persisted into the R8 stage, where seeds exhibited increased concentrations of total sugars, reducing sugars (fructose and glucose), and sucrose, accompanied by a ∼35% reduction in starch (Fig. 1h). Transcriptional analysis of central metabolic pathways revealed that sucrose metabolism genes, including sucrose synthase (*SuSy*), phosphoglucose isomerase (*PGI*), and phosphoglucomutase (*PGM*), were upregulated (Fig. 1i). Conversely, transcripts associated with starch biosynthesis, such as soluble starch synthases and branching/debranching enzymes, were broadly downregulated (Fig. 1i), aligning with the compromised AGPase activity and starch deficit. Surprisingly, the double mutants exhibited a distinct carbon partitioning trade-off at the R8 stage, characterized by significantly increased protein content accompanied by markedly reduced oil content relative to HC6 (Figure S5). Metabolic profiling revealed that this shift was associated with the accumulation of upstream precursors, including pyruvate, acetyl-CoA, malate, and amino acids such as lysine, aspartate, threonine, isoleucine, and methionine content (Figure S5). This metabolite profile reflects a blockage in fatty acid flux, consistent with the downregulated expression of core biosynthetic genes such as *GmMCAT1*, *GmKASII, and GmKASIII*, along with master regulators including *GmWRI1*, *GmCG1*, and *GmOLEO1* (Figure S6). Collectively, these data indicate that the repression of the lipid biosynthetic program channels carbon flux away from oil production toward amino acid synthesis and protein accumulation.

**Figure 1:**
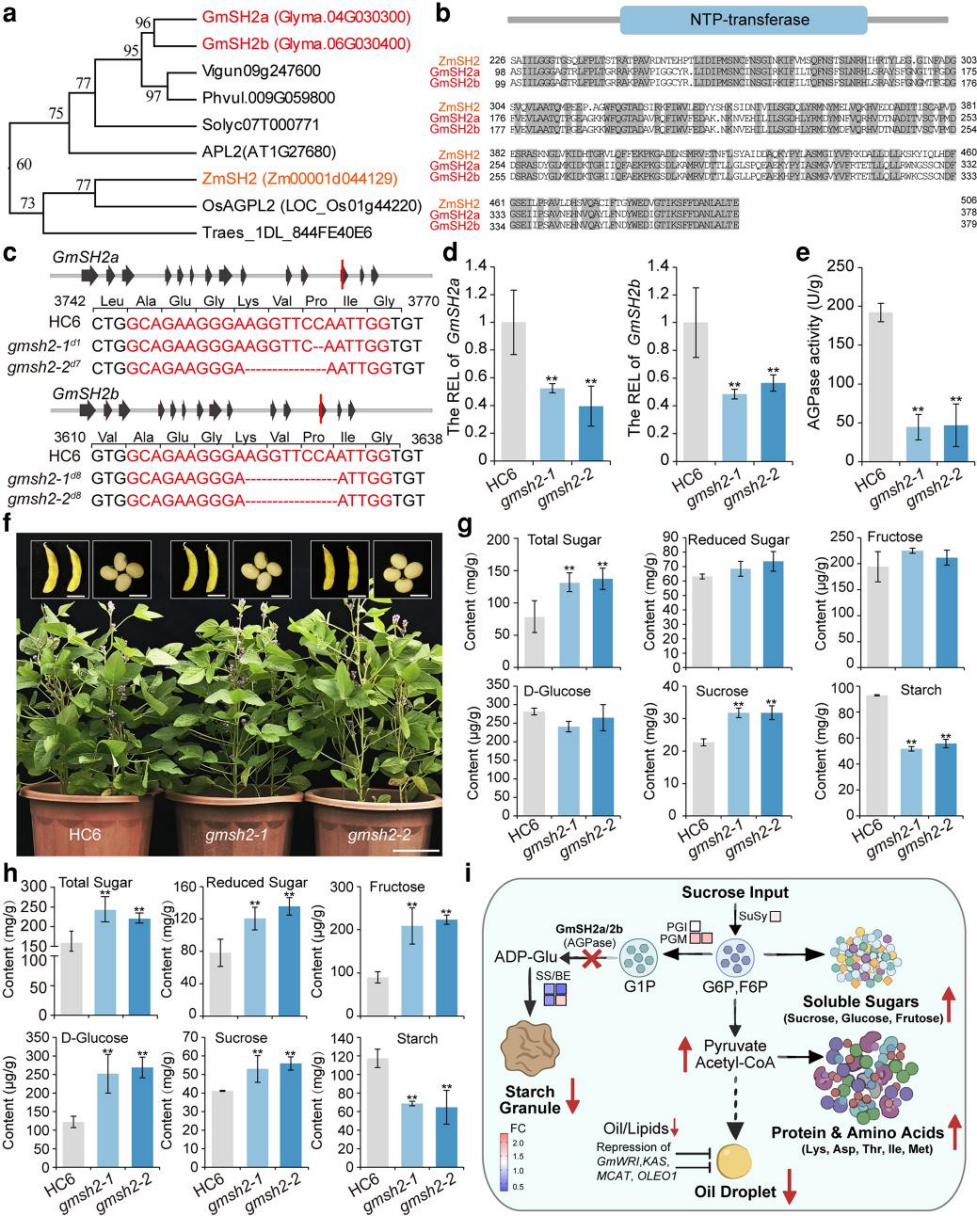
Mutation of *GmSH2* enhances soluble sugar content in soybean. (a) Phylogenetic analysis of SH2 proteins across different species. The phylogenetic tree was constructed using the Neighbor-Joining method. Soybean GmSH2a and GmSH2b are in red font. Maize ZmSH2 are in orange font. (b) Domain structure and sequence conservation of GmSH2 proteins. The upper panel shows a schematic representation of the domain architecture of SH2 protein. The conserved nucleotidyl transferase (NTP_transferase) domain is highlighted by a blue box, and the gray line represents the protein backbone. The lower panel displays the multiple sequence alignment of the NTP-transferase domains of GmSH2a, GmSH2b, and ZmSH2 proteins. Similar amino acid residues are shaded in gray. (c) Molecular characterization of *gmsh2* double mutants. Schematic representation of the CRISPR/Cas9-induced mutations in the *gmsh2* double mutants. The target sequences and specific indels are shown. In the gene structure schematics, gray lines and black block arrows denote introns and exons, respectively, while vertical red lines indicate Cas9 cleavage sites. In the sequence alignments, red font highlights the sgRNA target sequences, and red dashes represent nucleotide deletions in the mutant alleles. (d) Transcriptional analysis of *GmSH2a* and *GmSH2b* expression in *gmsh2* mutant. *GmActin* was used as the internal control, and the relative expression level (REL) of HC6 was set arbitrarily to 1. Data are means ± SD (n = 3). Asterisks indicate significant differences (**P < 0.01, Student's t-tests). (e) ADP-glucose pyrophosphorylase (AGPase) activity in both HC6 and *gmsh2* mutants. (f) Phenotypic comparison between HC6 and *gmsh2* mutants. Representative images of whole plants and pods at the R6 stage, and seeds at the R8 stage. Scale bars: whole plants, 10 cm; pods, 2 cm; seeds, 1 cm. (G and (h) Soluble sugar (total sugars, reducing sugars, fructose, glucose, and sucrose) and starch contents in seeds of HC6 and *gmsh2* mutants at the R6 (g) and R8 (h) stages. (i) Proposed model illustrating metabolic repartitioning in *gmsh2* mutant seeds. Loss of *GmSH2* function blocks starch synthesis, restricting ADP-glucose availability. This leads to an accumulation of upstream soluble sugars and glycolytic precursors. Consequently, carbon flux is redirected, channeling precursors (pyruvate, acetyl-CoA) towards enhanced amino acid and protein synthesis, while simultaneously repressing the lipid biosynthesis program (*GmWRI1a*, *GmKAS*, *GmMCAT*, *GmOLEO1*), resulting in a high-protein, low-oil phenotype in mature seeds. Purple and red squares represent fold changes (FC) in gene expression and sugar/starch content, respectively, in the *gmsh2-2* mutant relative to HC6. (SuSy: Sucrose synthase; invertase: β-fructofuranosidase; HK: Hexokinase; PGI: Phosphoglucose isomerase; PGM: Phosphoglucomutase; UGPase: UDP-glucose pyrophosphorylase; AGPase: ADP-glucose pyrophosphorylase; SS/BE: starch synthase/branching enzyme; G1P: glucose-1-phosphate; G6P: glucose-6-phosphate; F6P: fructose-6-phosphate; Asp: Aspartic acid; Lys: lysine; Thr: threonine; Ile: isoleucine; Met: methionine). Data are means ± SD (n = 3). Asterisks indicate significant differences (**P < 0.01, Student's t-tests).

Together, these data suggest that the loss of *GmSH2a*/*2b* limits ADP-glucose availability, thereby restricting starch biosynthesis and redirecting carbon flux towards soluble sugars, a metabolic shift further corroborated by the concurrent reduction in lipid biosynthesis. Furthermore, we introduced the *sh2* mutations into “Maodou 64”, an elite vegetable soybean cultivar (Figure S7a). The F2 segregating population enabled the isolation of distinct single and double *sh2* mutant lines (Figure S7b, c). Notably, the segregants harboring the double mutation exhibited significantly higher sugar accumulation than those with single mutations (Figure S7d), providing genetic resources for the development of supersweet Maodou cultivars. This work highlights the broader potential of genome editing to fine-tune sugar metabolism in legumes. Future research could extend these strategies to other crops, enabling the engineering of customized sugar profiles and contributing to flavor-oriented, sustainable crop improvement.

## Supplementary Material

kiag181_Supplementary_Data

## Data Availability

The data underlying this article are available in the article.
