# Supplementary material for: CRISPR/Cas9-mediated targeted editing of GmSH2 enhances sugar accumulation in vegetable soybean
Source: Plant Physiol. 2026 May 18;201(1):kiag181. doi: 10.1093/plphys/kiag181 (PMC13181402; doi:10.1093/plphys/kiag181)
Supplement: kiag181_Supplementary_Data [file kiag181_supplementary_data.pdf]

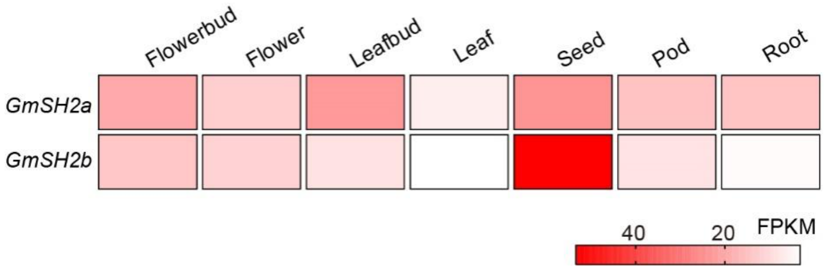

Fig. S1 Expression patterns of *GmSH2a* and *GmSH2b* in soybean, as retrieved from the Soytfbase database (http://www.soytfbase.cn/). FPKM, fragments per kilobase of transcript per million mapped reads.

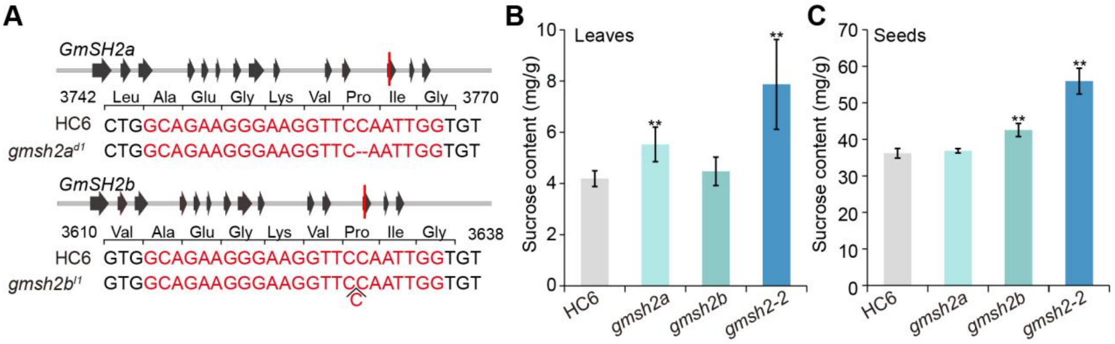

Fig. S2 Molecular characterization and sucrose analysis of *gmsh2* single and double mutants. (A) Schematic representation of the CRISPR/Cas9-induced mutations in the *gmsh2a* and *gmsh2b* single mutants. Genomic sequences and exons are indicated by gray lines and black block arrows, respectively, with red vertical lines marking Cas9 cleavage sites. The sgRNA target sequences (red text) and specific modifications (red dashes for deletions; red letter with caret for insertion) are shown below. (B and C) Sucrose contents in leaves (B) and seeds (C) of HC6, single mutants (*gmsh2a* and *gmsh2b*), and the double mutant (*gmsh2-2*). Data are means ± SD (n = 3). Asterisks indicate significant differences (**$P < 0.01$, Student's t-tests).

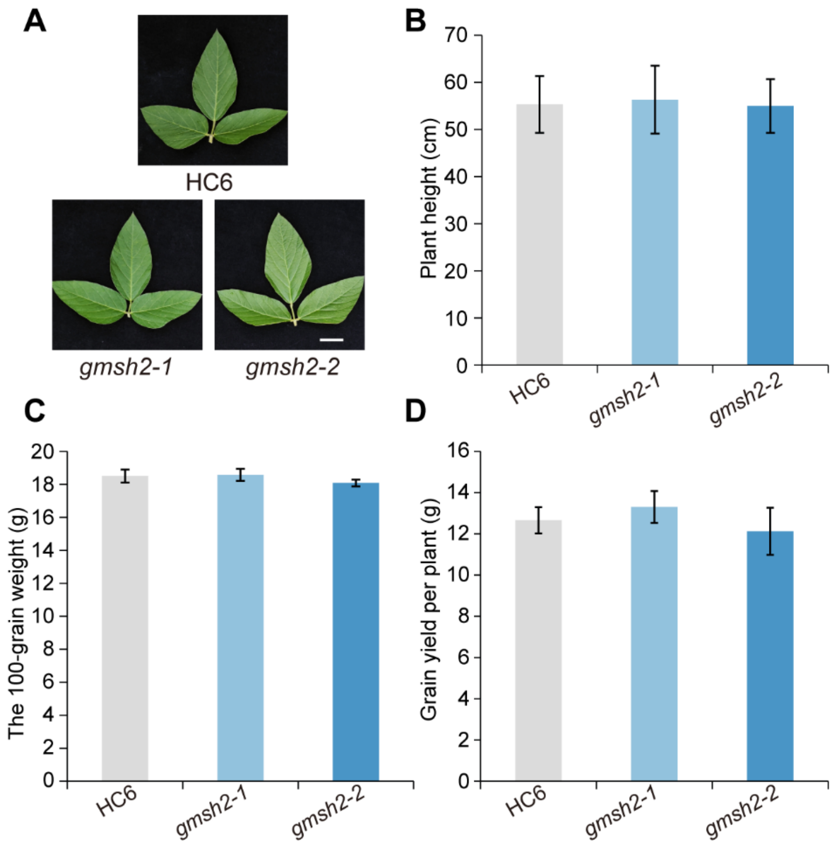

Fig. S3 Phenotypic characterization of *gmsh2* double mutants. (A) Representative images of leaf phenotypes of HC6 and mutant lines. Scale bar: 2 cm. The scale bar applies to all images in this panel. (B–D) Statistical analysis of plant height (B), 100-grain weight (C), and grain yield per plant (D). Data are means ± SD (n = 10 for plant height; n = 5 for 100-grain weight; n = 3 for grain yield).

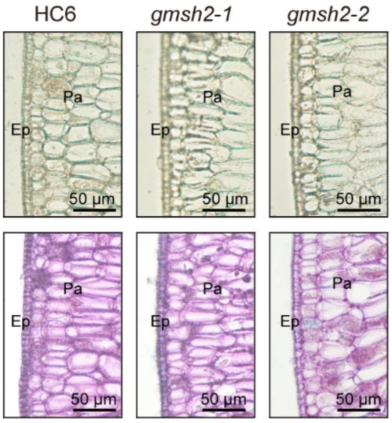

Fig. S4 Histological comparison of cotyledon cells in imbibed soybean seeds between HC6 and *gmsh2* mutants. Seeds were imbibed for 4 hours (until fully hydrated), decoated, and transversely sectioned (15 μm thickness) using a vibratome to preserve cotyledon structure. Sections were stained with Toluidine Blue. Ep: Epidermal cells (single layer of rectangular cells at the cotyledon edge); Pa: Parenchyma cells (large, polygonal cells filling the cotyledon interior, responsible for nutrient storage).

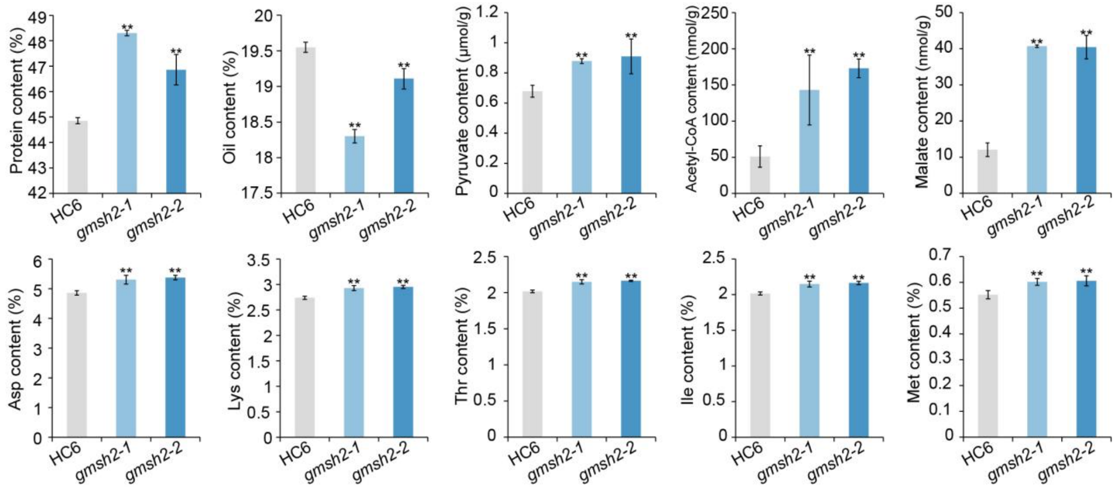

Fig. S5 Seed composition and metabolite analysis of HC6 and *gmsh2* mutants at the R8 stage. Contents of protein, oil, and amino acids (aspartic acid [Asp], lysine [Lys], threonine [Thr], isoleucine [Ile], and methionine [Met]) were measured using a Perten Near-Infrared Spectrum Analyzer. Metabolic intermediates, including pyruvate, acetyl-CoA, and malate, were quantified using commercial assay kits. Data represent means ± SD (n = 5). Asterisks indicate significant differences compared to HC6 (**$P < 0.01$ by Student's t-test).

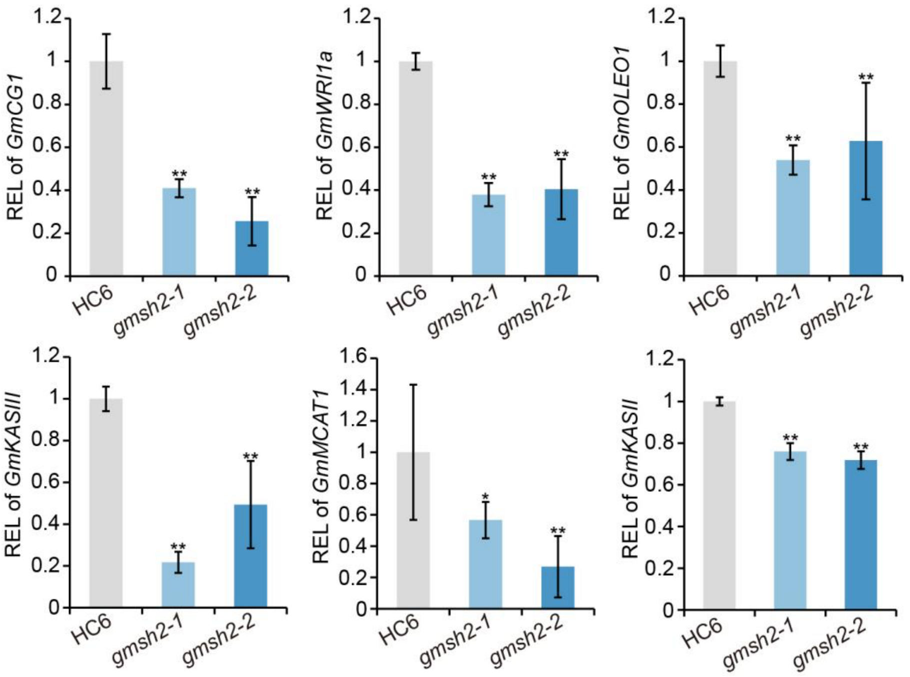

Fig. S6 Transcriptional analysis of genes associated with oil content in HC6 and *gmsh2* mutants. Relative expression levels (REL) were determined by RT-qPCR. *GmActin* was used as the internal control, and the expression level in HC6 was set to 1. Data represent means ± SD (n = 3). Asterisks indicate significant differences compared to HC6 (*$P < 0.05$, **$P < 0.01$ by Student's t-test).

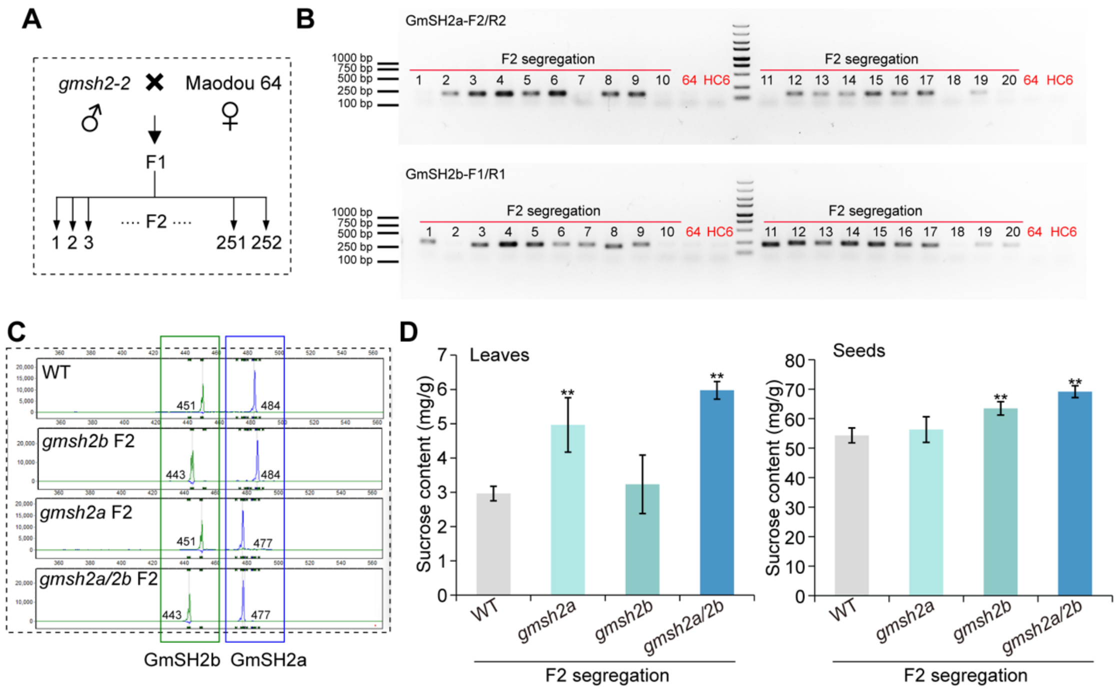

Fig. S7. Genetic segregation and sucrose accumulation in *gmsh2* mutant lines derived from 'Maodou 64'. (A) Schematic representation of the cross between the *gmsh2-2* mutant and 'Maodou 64', followed by self-pollination of F1 plants to generate the F2 segregating population. (B) PCR-based genotyping of F2 progeny using specific primers for *GmSH2a* (F2/R2) and *GmSH2b* (F1/R1). (C) Capillary electrophoresis profiles showing indel detection in F2 individuals. The green and blue boxes highlight the amplicons corresponding to the *GmSH2b* and *GmSH2a* loci, respectively. Peaks at 451 bp and 484 bp represent wild-type alleles of *GmSH2b* and *GmSH2a*, respectively, while mutant alleles are distinguished by fragment size shifts (e.g., 443 bp and 477 bp). (D) Sucrose content in leaves and seeds of wild-type (WT) and F2 segregants with distinct *gmsh2* genotypes. WT refers to F2 segregants lacking mutations. Data represent means ± SD (n = 3). Asterisks indicate significant differences compared to the WT control (*$P < 0.01$; Student's t-test).

Supplementary Table S1. listed primers used in this study.

Primer name	Forward primer (5'-3')	Reverse primer (5'-3')	Purpose
Glyma.04G032600	AGCTCTGGAGAAGTTTGCCC	GCTGCCCATTAGTTCCAGGT	qRT-PCR
Glyma.03G042000	GGACACCCAGACCCCAATTT	ACCATCAGAAGCAGCACCAA	qRT-PCR
Glyma.03G046100	TCTGGGAAGTTTTGGCCTGG	AGTGAGGACTGCAGCTTGAC	qRT-PCR
Glyma.02G240400	TCGTGTCAATTCATGGCGGA	CTGGGAGGGTAAATGCAGCA	qRT-PCR
Glyma.02G241100	CTCGTCAGCCGCTATCTCAG	AGAACCATCAGGAGTTGGCG	qRT-PCR
Glyma.03G192300	CAACGCAGTGCTACAGGCATTAC	AGAAGATCTCCCACACACCAAAT	qRT-PCR
Glyma.06G310900	TAGAAGAGAACTCATGCGTTGCC	TACACCGAGGAGATTTTTCCGTC	qRT-PCR
Glyma.01G096700	GGAGCATTCAACGTAGAAATCAG	TTATGTCCCAAAACACCTAGCCA	qRT-PCR
Glyma.04G235200	GCTACTTCACAAGACGGACCCTT	AAGCCAACGAGATAGCCAAAATC	qRT-PCR
GmSH2a	TGCCACTAAGATTATTGGGACACT	GAGAGAGAGAGAGAGTGAAGGGA	qRT-PCR
GmSH2b	TGCACCTTTAGATTAACTTAATCCCA	AACAGTGCATTCCTACCCCG	qRT-PCR
GmCG1	AGCAACACGGTGAGAAGGAG	CCGTGTTTTTCCTCCTTGCG	qRT-PCR
GmOLEO1	AGGCGCGTGAGATCAAGGACTATG	GCGTGCACACGATTAAGAAGCC	qRT-PCR
GmWRI1a	ACTTGGTGGGCATGTTTGATAGTG	AGTCTCATCACCAGGTTGAGTGC	qRT-PCR
GmKASIII	ATGCAGTTGCCACTCGGTTA	CCTTACCACTTCGAACCGCT	qRT-PCR
GmKASII	TTGTGGAGGCTCAGATGCTG	ACGGTTAATGTCCCAAGGGC	qRT-PCR
GmMCAT1	AGTGCTGGAATCCAAGGCAA	TCTGTTGTTGCCAATGCTGC	qRT-PCR
GmActin	CGGTGGTTCTATCTTGGCATC	GTCTTTCGCTTCAATAACCCTA	qRT-PCR
GmSH2a-F2/R2	AGTATGAAATTGCATCTCTGCTGGCT GAAGGGAAATTGG	CTAGGATCCATATACAAGGG	Mutation-specific molecular marker
GmSH2b-F1/R1	GTATGAAATTGCATCTCTGGTGGCAG AAGGGAATTGGTGTCACGG	AACCGAAATCAGAAATGC	Mutation-specific molecular marker

Supplementary Materials and Methods

Plant materials and growth conditions

The soybean cultivar "Huachun No.6" (HC6) was utilized for the purpose of this study. The plants were cultivated in a growth chamber under controlled conditions, with a photoperiod of 16 hours of light and 8 hours of darkness, and a temperature range of 24-26 °C. The relative humidity was maintained at 40% ± 10%.

Plasmid construction and the generation of transgenic plants

To generate the *gmsh2* mutant using CRISPR/Cas9 technology, guide RNAs (gRNAs) targeting a conserved region within exon 12 shared by both *GmSH2a* and *GmSH2b* were designed using the CRISPR-P webtool (http://crispr.hzau.eduau.edu.cn). Plasmid construction and transgenic plant generation were performed, following established protocols (Li et al., 2024).

Sample collection, RNA isolation, and quantitative real-time PCR (qRT-PCR) analysis

Developing soybean seeds at reproductive stages R6-R7 were harvested from (HC6 and *gmsh2* mutant plants, immediately frozen in liquid nitrogen, and stored at -80°C until RNA extraction. RNA extraction was performed using the FastPure Plant Total RNA Isolation Kit (Vazyme, China). The first-strand cDNA was synthesized from total RNA that had been treated with DNase using the Hiscript III Reverse Transcriptase kit (Vazyme, China). qRT-PCR was conducted in 96-well optical plates using a Roche Light Cycle 96 instrument and ChamQ Universal SYBR qPCR Master Mix (Vazyme, China). Relative expression levels (REL) of the control sample were arbitrarily assigned a value of 1. The primers utilized in this study could be found in Supplementary Table S1.

AGPase activity, soluble sugar, and starch content analysis

Developing soybean seeds at reproductive stages R6, R7, and R8 were harvested from (HC6 and *gmsh2* mutant plants, immediately frozen in liquid nitrogen, and stored at -80°C until analysis. The AGPase activity and starch content were measured using the AGPase Activity Assay Kit and Starch Content Assay Kit (Beijing Boxbio Science & Technology Co..Ltd.). The Pyruvate content (μmol/g), Acetyl-CoA content (nmol/g), and Malate content (nmol/g) were determined using commercial assay kits according to the manufacturer's protocols (Beijing Boxbio Science & Technology Co..Ltd.). For soluble sugar extraction, Frozen seed tissues (~100 mg fresh weight) were finely ground in liquid nitrogen, extracted in 3 mL of 80% (v/v) ethanol at 85°C for 30 min with constant agitation, centrifuged at 12,000 × g for 10 min, and the supernatant was collected. The pellet was re-extracted twice with 3 mL of 80% ethanol. The combined supernatants were incubated with powdered activated charcoal (~2% w/v) on a shaker at room temperature for 12 hours to adsorb pigments. The mixture was then centrifuged to remove charcoal. Combined supernatants were evaporated to dryness and reconstituted in 400 μL of ultrapure water, and then analyzed for total

sugar, reduced sugar, fructose, D-glucose, and sucrose contents using the specific sugar assay kit from the same manufacturer.

Histological analysis of soybean cotyledons

Seeds were imbibed in distilled water for 4 h at room temperature until fully hydrated. Following imbibition, the seed coats were carefully removed to expose the cotyledons. The decoated seeds were immediately subjected to transverse sectioning using a vibrating microtome (vibratome). Cross-sections with a uniform thickness of 15 μm were collected to preserve the intact cellular structure of the cotyledons. The resulting sections were stained with Toluidine Blue O to facilitate the visualization and comparison of cellular structures, particularly the epidermal (Ep) and storage parenchyma (Pa) cells.

Protein and oil content analysis

The Protein content (%), oil content (%), Aspartic acid (Asp) content (%), Lysine (Lys) content (%), Threonine (Thr) content (%), Isoleucine (Ile) content (%), and (J) Methionine (Met) content (%) were measured using the Perten Near-Infrared Spectrum Analyzer.

Mutation-specific molecular marker designing

Mutation-specific genotyping was performed using the derived Cleaved Amplified Polymorphic Sequences (dCAPS) method. Primers containing deliberate 2 bp mismatches were designed using the online tool (http://helix.wustl.edu/dcaps/). For the *GmSH2a* gene in the *gmsh2-2* mutant line and the *GmSH2b*, PCR products were directly electrophoresed without digestion. All amplification products were resolved by agarose gel electrophoresis (1% for undigested PCR products) and visualized by ethidium bromide staining.

SNP detection by Capillary Electrophoresis

Genomic DNA was extracted from young leaves of F_2 individuals using the CTAB method. Allelic variation at the *GmSH2a* and *GmSH2b* loci was detected by PCR amplification followed by capillary electrophoresis. Gene-specific primers flanking the target SNP sites were designed with a 5′-tail containing the forward sequence (5′-CACGACGCTCTTCCGATCT-3′) on the forward primer to enable fluorescent labeling. Fragment sizes were determined using GeneMapper® software v5.0. Wild-type alleles of *GmSH2b* and *GmSH2a* produced amplicons of 451 bp and 484 bp, respectively, while mutant alleles harboring deletions or insertions were identified by shifted peak sizes (e.g., 443 bp and 477 bp).
